# Roles of Gut Microbiota in the Regulation of Hippocampal Plasticity, Inflammation, and Hippocampus-Dependent Behaviors

**DOI:** 10.3389/fcimb.2020.611014

**Published:** 2021-01-27

**Authors:** Wen Tang, Zhaoyou Meng, Ning Li, Yiyan Liu, Li Li, Dongfeng Chen, Yang Yang

**Affiliations:** ^1^Department of Gastroenterology, Daping Hospital, Army Medical University, Chongqing, China; ^2^Department of Neurology, Xinqiao Hospital, Army Medical University, Chongqing, China; ^3^College of Basic Medicine, Army Medical University, Chongqing, China; ^4^Department of Gastroenterology, The First People’s Hospital in Chongqing Liangjiang New Area, Chongqing, China

**Keywords:** gut microbiota, hippocampus, learning and memory, senile plaque, inflammation, Alzheimer’s disease

## Abstract

The study of the gut microbiota-brain axis has become an intriguing field, attracting attention from both gastroenterologists and neurobiologists. The hippocampus is the center of learning and memory, and plays a pivotal role in neurodegenerative diseases, such as Alzheimer’s disease (AD). Previous studies using diet administration, antibiotics, probiotics, prebiotics, germ-free mice, and fecal analysis of normal and specific pathogen-free animals have shown that the structure and function of the hippocampus are affected by the gut microbiota. Furthermore, hippocampal pathologies in AD are positively correlated with changes in specific microbiota. Genomic and neurochemical analyses revealed significant alterations in genes and amino acids in the hippocampus of AD subjects following a remarkable shift in the gut microbiota. In a recent study, when young animals were transplanted with fecal microbiota derived from AD patients, the recipients showed significant impairment of cognitive behaviors, AD pathologies, and changes in neuronal plasticity and cytokines. Other studies have demonstrated the side effects of antibiotic administration along with the beneficial effects of probiotics, prebiotics, and specific diets on the composition of the gut microbiota and hippocampal functions, but these have been mostly preliminary with unclear mechanisms. Since some specific gut bacteria are positively or negatively correlated to the structure and function of the hippocampus, it is expected that specific gut bacteria administration and other microbiota-based interventions could be potentially applied to prevent or treat hippocampus-based memory impairment and neuropsychiatric disorders such as AD.

## Introduction

The human microbiome is established early in life, and consists of approximately 3.8 × 10^13^ symbiotic microorganisms (Lukiw, 2016; O’Hagan et al., 2017). In the gastrointestinal tract, the colonized gut microbiota is a complex and dynamic community of microorganisms that can communicate with the host to influence the brain and behavior (Liang et al., 2015; Hu et al., 2020). Under normal conditions, aging is associated with changes in higher brain functions such as learning and memory, as well as dysbiosis in the gut microbiome (Daulatzai, 2014; Distrutti et al., 2014). One hundred years ago, the Nobel Prize winner Elie Metchnikoff proposed that cognitive decline and senility might be delayed by manipulating the intestinal microbiome with host-friendly bacteria (Scott et al., 2017). However, no significant progress showing that the bacterial constituents of the gut microbiota can influence brain function has been made over the past decade (O’Hagan et al., 2017). The term gut-microbiota-brain axis or gut-brain-axis is used to describe the relationship between the gut and the brain (Bienenstock and Collins, 2010).

The hippocampus, consisting of the cornu ammonis (CA) 1, CA2, CA3, dentate gyrus (DG), and subiculum, is the center of learning and memory (Lisman et al., 2017; Hainmueller and Bartos, 2018). Interestingly, although engrams (memory traces) in CA1 and CA2 do not stabilize over time, reactivation of engrams in the DG can induce recall of artificial memories even after weeks (Hainmueller and Bartos, 2018). Moreover, the hippocampus has also been implicated in depression and anxiety, and hippocampal neurogenesis has been implicated in cognitive processes (Toda et al., 2018). Since the gut microbiota has been shown to play a role in the pathology of Alzheimer’s disease (AD) and other memory disorders, we reviewed the current progress on the gut microbiota’s influence on the structure and function of the hippocampus and hippocampus-based learning and memory.

## Imbalanced Gut Microbiota in Alzheimer’s Disease Subjects and Model Animals

AD is the most common neurodegenerative disorder, ultimately resulting in dementia, and the hippocampus is one of the affected brain regions (Moodley and Chan, 2014). Several clues from human fecal studies have shown that gut microbiota composition is different between AD patients and healthy controls (HCs). For example, AD patients showed lower abundance of *Eubacterium* but higher abundance of *Escherichia/Shigella* (Cattaneo et al., 2017), along with obvious changes in *Bacteroides*, *Actinobacteria*, *Ruminococcus*, *Lachnospiraceae*, and *Selenomonadales* (Zhuang et al., 2018). Other studies showed that among AD patients, patients with amnestic mild cognitive impairment, and HCs, the fecal microbial diversity was changed, showing a reduced proportion of phylum *Firmicutes* but enriched *Proteobacteria*. These results indicated that distinct microbial communities, especially enriched *Enterobacteriaceae*, were associated with AD (Zhuang et al., 2018; Liu et al., 2019). Furthermore, gene-targeted analysis of human gut microbiota in AD fecal samples found some unique gut bacterial sequences that were rarely seen in controls, highlighting the significant difference in the gut microbial genotypes between the AD patients and healthy human populations (Paley et al., 2018).

AD model rodents have been frequently used to explore alterations in the gut microbiota in AD. In the feces of AD mice, the microbiota composition and diversity were changed, with short-chain fatty acid composition (Zhang et al., 2017) and the amount of trypsin reduced when compared to wild type (WT) mice (Brandscheid et al., 2017). Additionally, the composition and diversity of the gut microbiota changed greatly with aging and AD pathology. Impaired spatial memory appeared in 6-month-old APP/PS1 AD model mice and was further aggravated in the 8-month-old mice. This was consistent with the accumulation in amyloid plaque and the remarkable shift in gut microbiota compared to WT mice. The abundance of *Helicobacteraceae*, *Desulfovibrionaceae*, *Odoribacter*, and *Helicobacter* increased significantly, while that of *Prevotella* decreased significantly (Shen et al., 2017). At 3 months of age, the fecal bacterial profiles did not show significant differences between the AD mice and control mice; however, at 6 months, the abundance of *Turicibacteriaceae* and *Rikenellaceae* increased in both groups, and an increase in *Proteobacteria* abundance was seen in AD mice after 6 months, particularly that of the genus *Sutterella* (*Betaproteobacteria*); the inflammation-related family *Erysipelotrichaceae* was more abundant in 24-month-old AD mice than in WT mice (Bauerl et al., 2018). These results indicated that AD pathology shifted gut microbiota composition towards an inflammation-related bacterial profile during aging, and suggested that these changes could contribute to disease progression and severity (Bauerl et al., 2018). Importantly, recent studies showed that when the gut microbiota from AD patients was transplanted into AD mice, the recipient mice showed more severe cognitive impairment and activated microglia in the hippocampus, and these effects could be effectively inhibited by transplantation of healthy human gut microbiota (Shen et al., 2020).

**Figure 1:**
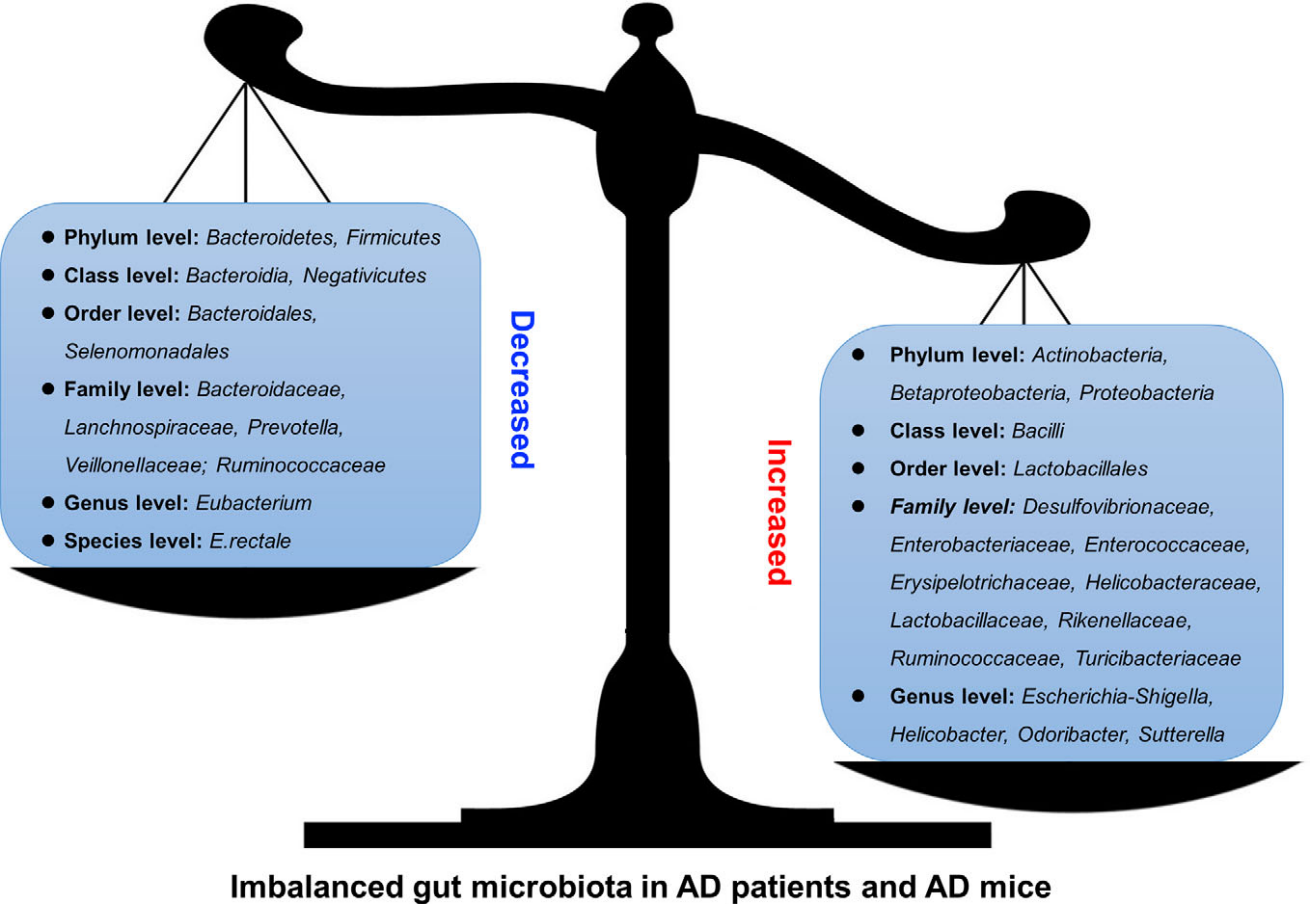
Altered gut microbiota in AD patients and model mice. Some differences have been noticed regarding the changes of gut microbiota in AD patients or mouse models. For example, *Bacteroidetes* and *Firmicutes* decreased (left) at Phylum level while *Actinobacteria*, *Betaproteobacteria*, and *Proteobacteria* increased (right).

Thus, in both AD patients and AD model animals, significant changes in the gut microbiota have been reported, some of which increased while others decreased (**Figure 1** and **Table 1**), indicating that manipulation of the gut microbiota may be a promising intervention for the prevention or treatment of AD.

**Table 1:** Altered gut microbiota in Alzheimer’s disease (AD) patients and mice.

Object	Increased/enriched	Decreased	References
AD patients	*Escherichia-Shigella*	*Eubacterium, E. rectale*	(Cattaneo et al., 2017)
AD patients	*Proteobacteria, Actinobacteria, Enterobacteriaceae*	*Firmicutes*	(Liu et al., 2019)
AD patients	*Actinobacteria, Bacilli, Lactobacillales, Ruminococcaceae, Enterococcaceae, Lactobacillaceae*	*Bacteroidetes, Negativicutes, Bacteroidia, Bacteroidales, Selenomonadales, Lanchnospiraceae, Bacteroidaceae, Veillonellaceae*	(Zhuang et al., 2018)
AD mice	*Helicobacteraceae, Desulfovibrionaceae, Odoribacter, Helicobacter*	*Prevotella*	(Shen et al., 2017)
AD mice	*Turicibacteriaceae, Rikenellaceae, Proteobacteria, Sutterella, Betaproteobacteria, Erysipelotrichaceae*	*Ruminococcaceae*, *Lachnospiraceae*. *Bacteroidaceae*	(Bauerl et al., 2018)

## Hippocampal Neurochemicals and Neuroplasticity are Regulated by the Gut Microbiota

Changes in neurochemicals form the basis of structural and functional plasticity of the hippocampus. An early analysis of the cerebral metabolome revealed that the concentrations of 38 metabolites differed significantly between germ-free (GF) mice and WT mice, indicating that intestinal microbiota is closely related to brain health and disease and its functions, such as development, learning, memory, and behavior (Matsumoto et al., 2013). Kawase et al. reported that compared to specific pathogen-free (SPF) mice, hippocampal amino acids and neurochemicals in GF mice at postnatal week 7 were significantly changed, showing lower concentrations of L-Ala, l-Arg, l-Gln, l-Ile, l-Leu, l-Phe, l-Val, and GABA, but higher concentrations of Ser (Kawase et al., 2017). Another study showed that GF mice showed higher hippocampal levels of creatine, N-acetyl-aspartate, lactate, and taurine but lower levels of succinate than SPF mice (Swann et al., 2017). Furthermore, the hippocampus of GF mice showed an increase in synapse-promoting genes and markers of reactive microglia and synaptic density, all of which could be reversed by colonization with human *Bifidobacterium* species or conventional murine microbiota, indicating that *Bifidobacteria* are involved in the establishment of functional neural circuits in the hippocampus (Luck et al., 2020). Interestingly, one hippocampal microRNA (miRNA) study using GF, conventional, and GF colonized mice showed an increase in miR-294-5p expression in GF animals but normalized expression following colonization, indicating that the gut microbiota plays an important role in modulating small RNAs that influence hippocampal gene expression (Moloney et al., 2017). Similarly, one study showed that in the hippocampus of GF mice, 1355 lncRNAs were upregulated and 875 lncRNAs were downregulated. Further analysis revealed that most of their target genes were highly associated with cardiac hypertrophy, nuclear factors of activated T cells, gonadotropin-releasing hormone, calcium, and cAMP-response element-binding protein (CREB) signaling pathways (Zhou et al., 2020).

The brain-derived neurotrophic factor (BDNF) regulates activity-dependent synaptic plasticity and psychiatric disorders (Bjorkholm and Monteggia, 2016; Leal et al., 2017), while CREB regulates genes related to neuronal differentiation, synaptic plasticity, learning, and memory (Sharma et al., 2019). Studies have shown that both hippocampal BDNF and CREB are regulated by the gut microbiota. The anticancer flavonoid quercetin, a secondary plant metabolite, has been shown to increase gut microbial diversity and relative abundance of *Glutamicibacter*, *Facklamia*, and *Aerocorrus*; increase hippocampal BDNF; and improve learning and memory (Lv et al., 2018). Zeng et al. used microarray analysis and revealed that the absence of the gut microbiota from birth was associated with decreased hippocampal CREB but an increase in phosphorylated CREB (pCREB), which could be restored by microbiota colonization in adolescence; hippocampal pCREB expression could be reduced by removal of the gut microbiota from SPF mice using antibiotics (Zeng et al., 2016). Additionally, oral administration of *Lactobacillus johnsonii CJLJ103*, a member of the human gut microbiota, may alleviate cholinergic memory impairment by increasing BDNF expression and pCREB in the hippocampi (Lee et al., 2018). Interestingly, gut microbiota-induced hippocampal BDNF expression might be mediated by the vagus nerve, since it could be regulated by subdiaphragmatic vagotomy (O’Leary et al., 2018). A recent study showed that when fecal microbiota transplantation (FMT) was conducted on aged and young rats, the young rats showed impairment in cognitive behavior, a decrease in dendritic spines and expression of BDNF, N-methyl-D-aspartate receptor NR1 subunit, and synaptophysin, but an increase in the expression of advanced glycation end products (AGEs) and receptors for AGEs. At the phylum level, FMT decreased the relative abundance of *Bacteroidetes*, while increasing the relative abundance of *Actinobacteria*. At the genus level, FMT rats showed lower levels of *Prevotella*, *Bacteroides*, *Parabacteroides*, and higher levels of *Sutterella* (Li et al., 2020).

Furthermore, studies have shown that the morphology and neurogenesis of the hippocampus are regulated by the gut microbiota. Convincing evidence comes from studies of GF animals. Luczynski et al. reported that compared to the control mice, GF mice showed significant hippocampal expansion with shorter pyramidal neurons, and less-branched, stubby mushroom- spines and granule cells (Luczynski et al., 2016). Indirectly, Val-Laillet et al. found that a Western diet (fat 33%, refined carbohydrate 49%) induced a decrease in microbiota activity and hippocampal neurogenesis but increased cell proliferation, higher working memory and reference memory scores, accompanied by a smaller hippocampal granular cell layer volume (Val-Laillet et al., 2017) Similarly, Möhle et al. found that antibiotics, which could severely deplete the intestinal microbiota, significantly decreased hippocampal neurogenesis (Mohle et al., 2016).

Probiotics, diets, and obesity also play roles in the regulation of the hippocampus, which might be mediated by the gut microbiota. Distrutti et al. reported that treatment of aged rats with VSL#3, a probiotic mixture comprising eight gram-positive bacterial strains, increased the abundance of *Actinobacteria* and *Bacteroidetes* and modulated the expression of CD11b (a marker for microglia), BDNF, syntaxin, and drebrin in the hippocampus (Distrutti et al., 2014). VSL#3 has also been shown to prevent diet-induced microbiota deficits by increasing the abundance of some taxa such as *Streptococcus*, *Lactobacillus*, and *Butyrivibrio*, which were decreased by the cafeteria (Caf) diet. Meanwhile, hippocampal-dependent place tasks were also regulated by these treatments (Beilharz et al., 2018). However, in the hippocampus, the Caf diet increased the expression of many neuroplastic genes and serotonin receptor 5-HT1A, which are the best predictors of place memory, and are related to the microbiota principal component (PC) 1 (Beilharz et al., 2018). For obese humans, hierarchical clustering with magnetic resonance imaging analysis revealed a specific gut microbiota-brain map profile, and the Shannon index was linked to R2* and fractional anisotropy of the hippocampus (Fernandez-Real et al., 2015). Moreover, changes in waist circumference in obese humans are associated with iron deposition in the hippocampus, and these changes are linked to shifts in the gut microbiome (Blasco et al., 2017).

**Figure 2:**
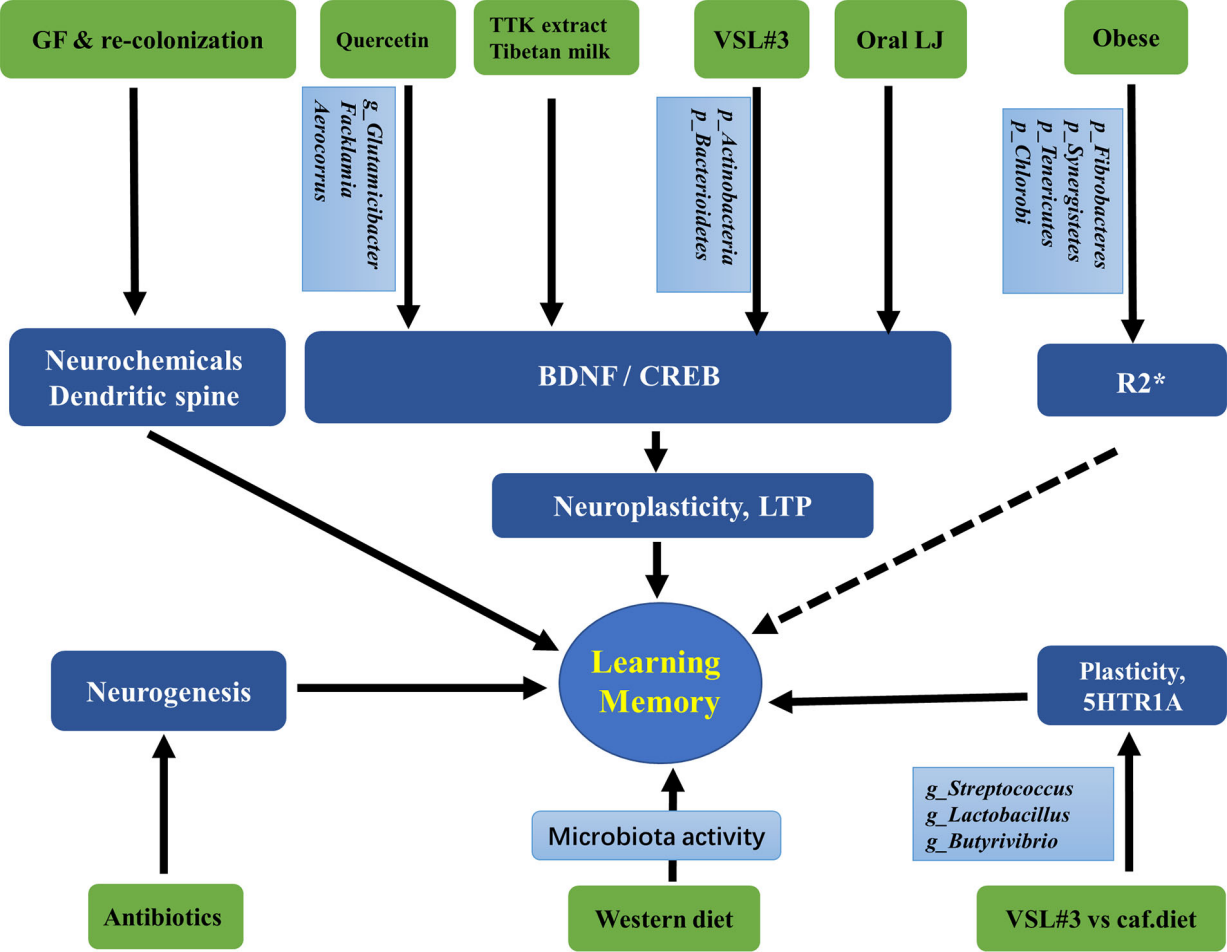
Associations between gut microbiota and the hippocampus-dependent plasticity and behaviors. GF, Germ free; LJ, *Lactobacillus johnsonii CJLJ103*; VSL3#, a probiotic mixture comprising 8 Gram-positive bacterial strains; caf, cafeteria; LTP, long-term potentiation, the cellular mechanism of synaptic plasticity; TTK, Tetragonia tetragonioides Kuntze extract; R2*, a validated magnetic resonance imaging (MRI) marker of brain iron content which can be rapidly measured under clinical conditions. The taxonomic group of bacteria: Phylum, Class, Order, Family, Genus, Species, were marked with p, c, o, f, g, s.

Taken together, the current findings suggest that the gut microbiota can be regulated by antibiotics, probiotics, diets, and obesity. They further affect hippocampus-dependent behaviors by acting on neurochemicals, neurotrophic factors, transcriptional factors, neurogenesis, and plasticity of pyramidal and granular cells. These findings are summarized in **Figure 2** and **Table 2**.

**Table 2:** Associations between gut microbiota and the hippocampal-dependent behaviors.

Treatment	Related microbiota	Hippocampal target	Behavior	References
quercetin	*Glutamicibacter*, *Facklamia*, *Aerocorrus*	BDNF	learning and memory	(Lv et al., 2018)
GF mice and colonization		CREB, pCREB	anxiety-related and passive behaviors	(Zeng et al., 2016)
oral administration	*Lactobacillus johnsonii CJLJ103* (LJ)	BDNF, pCREB	cholinergic memory	(Lee et al., 2018)
FMT	*Prevotella, Bacteroide, Parabacteroides, Sutterella*	dendritic spines, BDNF, NMDA receptor, synaptophysin, AGEs and receptor	cognitive behavior	(Li et al., 2020)
germ free		hippocampal expansion, neurons, dendritic spine		(Luczynski et al., 2016)
Western diet	microbiota activity	decreased neurogenesis, increased proliferation	working and reference memory	(Val-Laillet et al., 2017)
antibiotics		neurogenesis		(Mohle et al., 2016)
probiotic mixture VSL#3	*Actinobacteria* and *Bacterioidetes*	BDNF, neuronal plasticity, LTP, inflammation		(Distrutti et al., 2014)
probiotic mixture VSL#3 vs cafeteria diet	*Streptococcus*, *Lactobacillus*, *Butyrivibrio*	neuroplasticity, serotonin receptor (5HT) 1A	place memory	(Beilharz et al., 2018)
obese	*Fibrobacteres*, *Synergistetes, Tenericutes RA#, Chlorobi RA*	right hippocampus R2*		(Blasco et al., 2017)
obese	diversity	lowest R2*		(Fernandez-Real et al., 2015)

GF, germ free; FMT, fecal microbiota transplantation; LTP, Long-term potentiation; VSL#3, a probiotic mixture comprising 8 Gram-positive bacterial strains (Streptococcus thermophilus DSM24731, Bifidobacterium breve DSM24732, Bifidobacterium longum DSM24736, Bifidobacterium infantis DSM24737, Lactobacillus acidophilus DSM24735, Lactobacillus plantarum DSM24730, Lactobacillus paracasei DSM24733, Lactobacillus delbrueckii subspecies Bulgaricus DSM24734).

## Alterations in the Gut Microbiota Affect Hippocampus-Dependent Learning and Memory

Numerous studies have revealed that the gut microbiota may affect hippocampus-dependent learning, memory, and behavior. Probiotics regulate learning and memory through action on the gut microbiota. When old (15–17 months) mice were treated with a multi-species live *Lactobacillus* and *Bifidobacteria* mixture (*Lactobacillus acidophilus CUL60*, *L. acidophilus CUL21*, *Bifidobacterium bifidum CUL20*, and *B. lactis CUL34*), the spatial navigation, as shown by the results of a water maze, was moderately improved and the long-term object recognition memory was dramatically improved (O’Hagan et al., 2017). These results indicate that chronic dietary supplements with multi-species live microorganisms have beneficial effects on memory. Kobayashi et al. showed that oral administration of *Bifidobacterium breve strain A1 (B. breve A1*) to AD mice reversed the impaired behavior in a Y-maze test and the reduced latency in a passive avoidance test. Further gene profiling analysis revealed that *B. breve A1* administration suppressed the expression of hippocampal inflammation and immune-reactive genes that were induced by amyloid beta (Aβ) (Kobayashi et al., 2017). Additionally, in a mouse model of vascular dementia, *Clostridium butyricum* treatment was shown to increase the diversity of intestinal bacteria, improve spatial learning and memory dysfunction, and morphological changes in hippocampal granule cells. It also activated the BDNF-PI3K/Akt pathway in the hippocampus (Liu et al., 2015).

Plant extracts may affect learning and memory through action on the gut microbiota. In a d-galactose-induced aging mouse model, tuna oil administration restored the diversity of the gut microbiota, showing significant changes in 27 key operational taxonomic units; it also alleviated aging and memory deterioration and changed the expression of proteins related to synaptic repair and signal transduction (Zhang et al., 2018). Additionally, treatment of LW-AFC, an herbal medicine prepared from the traditional Chinese medicine LiuweiDihuang decoction, was given to senescence-accelerated mouse prone 8 (SAMP8) mice, which resulted in improvement of cognitive impairments including spatial learning and memory, active avoidance response, and object recognition memory capability. This was accompanied by significant changes in operational taxonomic units (OTUs; eight increased and 12 decreased) in the gut microbiota. Further examinations showed that there were seven OTUs significantly correlated with all three types of cognitive abilities (three negative and four positive correlations) at the order level, including *Bacteroidales*, *Clostridiales*, *Desulfovibrionales*, and *CW040* (Wang et al., 2016). Tetragonia tetragonioides Kuntze (TTK) extract was also shown to protect against short-term and special memory loss, which might involve the upregulation of the hippocampal pCREB/pAk/pGSK-3β pathway, expression of BDNF and CNTF, and cytokines such as TNF-α and IL-1β. These changes were accompanied by a decrease in *Clostridiales*, *Erysipelotrichales*, and *Desulfovibrionales* but an increase in *Lactobacillales* and *Bacteroidales* (Kim et al., 2020). Such cognition-improving effects were seen in Tibetan fermented milk-treated APP/PS1 AD mice, which showed an increase in intestinal microbial diversity and increased abundance of *Bacteroides, Faecalibacterium* spp. Mucispirillum, and *Ruminiclostridium;* cognitive function was negatively correlated with *Mucispirillum* abundance and positively correlated with *Muribaculum* and *Erysipelatoclostridium* abundance (Liu et al., 2020). These results are summarized in **Table 3**.

**Table 3:** Gut microbiota and hippocampus-dependent memory.

Treatment	Gut microbiota	Behavior	References
*Lactobacillus* and *Bifidobacteria* mixture	*Lactobacillus acidophilus CUL60*, *L. acidophilus CUL21*, *Bifidobacterium bifidum CUL20* and *B. lactis CUL34*	spatial navigation, long-term object recognition memory	(O’Hagan et al., 2017)
oral administration	*Bifidobacterium breve strain A1*	Y maze, passive avoidance	(Kobayashi et al., 2017)
	*Clostridium butyricum*	spatial learning and memory	(Liu et al., 2015)
tuna oil	microbiota diversity	memory	(Zhang et al., 2018)
LW-AFC	operational taxonomic units (*Bacteroidales*, *Clostridiales*, *Desulfovibrionales* and *CW040*)	spatial learning and memory, active avoidance, object recognition memory	(Wang et al., 2016)
Tetragonia tetragonioides Kuntze extract	decrease in *Clostridiales*, *Erysipelotrichales*, and *Desulfovibrionales* but increase in *Lactobacilales* and *Bacteroidales*	short-term and special memory	(Kim et al., 2020)
Tibetan fermented milk	*Bacteroides*, *Faecalibacterium* spp. Mucispirillum, *Ruminiclostridium; Muribaculum*, *Erysipelatoclostridium*	cognitive function	(Liu et al., 2020)

LW-AFC: an herbal medicine prepared from traditional Chinese medicine from LiuweiDihuang decoction.

## Gut Microbiota and Hippocampal Inflammation

Inflammation in the hippocampus is key to the vulnerability and recovery from psychiatric disorders. Several studies have reported that the gut microbiota may change the hippocampal inflammatory response and the related behaviors. For example, in obese mice, alterations in the gut microbiota could be ameliorated by *B. pseudocatenulatum CECT 7765* accompanied by reduced Toll-like receptor 2 (TLR2) protein or gene expression in the hippocampus (Agusti et al., 2018). An early study showed that exposure to magnesium deficient diet induced changes in gut microbiota composition that was positively correlated to the levels of hippocampal interleukin-6 (IL-6) (Winther et al., 2015). Beilharz et al. found that a diet with saturated fatty acid and sugar but lacking polyunsaturated fatty acid significantly impaired hippocampal-dependent place recognition memory accompanied by altered composition of gut microbes. Further analysis revealed that the strongest relationship was detected between hippocampal IL-1b, TLR4, PPARGC1A, PLA24GA, PTGES2, and microbiota PC2 or PC3 (Beilharz et al., 2016), indicating the existence of a gut-microbiota-hippocampal inflammation-behavior axis. Teasaponin, the major active component of tea, has been shown to attenuate gut microbiota alterations induced by a high-fat diet, prevent recognition memory impairment, and improve neuroinflammation deficits (indicated by levels of TLR4, MyD88, p-JNK, NF-κB, IL-1β, IL-6, and TNF-α) in the hippocampus (Wang et al., 2017). Furthermore, treatment of aged rats with VSL#3 induced a robust change in the composition of intestinal microbiota, with an increase in the abundance of *Actinobacteria* and *Bacteroidetes*; modulated expression of inflammatory genes, such as CD68 mRNA and CD11b mRNA in hippocampal slices; and decreased expression of markers of microglial activation (Distrutti et al., 2014).

The Gram-negative facultative anaerobe *B. fragilis*, which constitutes an appreciable proportion of the human gastrointestinal gut microbiome that secretes an unusually complex mixture of neurotoxins, including extremely proinflammatory lipopolysaccharides (LPS) (Zhao and Lukiw, 2018). Unexpectedly, Zhang et al. reported abundant LPS immunoreactivity in the AD-affected hippocampus, indicating that a major source of proinflammatory signals in the AD brain may originate from the gut microbiome due to intestinal mucosa barrier and blood-brain barrier dysfunction (Zhang et al., 2017). It has been shown that LPS-induced changes in *Firmicutes* commensals and depletion *Proteobacteria* opportunistic organisms were reversed to control levels by FMT in male rats, and LPS mice treated with FMT showed better spatial memory in behavioral tests (Li et al., 2018). A recent study by Mohammadi et al. showed that a probiotic formulation (*Lactobacillus helveticus R0052* and *Bifidobacterium longum R0175*) reversed LPS-induced elevation of both the circulating and hippocampal levels of proinflammatory cytokines, and attenuate the effect of LPS on memory (Mohammadi et al., 2019). Furthermore, LPS were shown to drive an NF-kB-miRNA-mediated deficiency in gene expression that contributes to alterations in synaptic architecture, synaptic deficits, amyloidogenesis, innate immune defects, and progressive inflammatory signaling, all of which are characteristics of AD-type neurodegeneration (Zhao and Lukiw, 2018).

Many factors are involved in the pathogenic gut microbiota-related systemic inflammation, due to increased LPS and proinflammatory cytokines, barrier dysfunction, and dysfunctional vago-vagal gut-brain axis (Daulatzai, 2014). The colitis mice showed impaired memory, increased fecal and blood levels of LPS, an increase in *Enterobacteriaceae*, but a decrease in *Lactobacillus johnsonii*. These changes in behaviors and LPS production could be induced by treatment with *E. coli* isolated from the feces of colitis mice accompanied with NF-κB activation and TNF-α expression as well as suppressed BDNF expression in the hippocampus of mice. However, all these changes could be reversed by treatment with *Lactobacillus johnsonii* (Jang et al., 2018). This was further demonstrated by oral administration of *Lactobacillus brevis OW38* to aged mice showing reduced LPS levels in colon fluid and blood and reduced ratio of *Firmicutes* to *Bacteroidetes* or *Proteobacteria* to *Bacteroidetes*, which was significantly higher in aged mice than in young mice. Treatment with OW38 in aged mice inhibited the expression of inflammatory markers (such as TNF and IL-1β) and NF-κB activation, and suppressed the expression of senescence markers (p16, p53, and SAMHD1) in the hippocampus of aged mice (Jeong et al., 2016). These results strongly demonstrated that gut microbiota disturbance could induce hippocampal inflammation and memory impairment. Moreover, it has been reported that when FMT is conducted, young recipient rats show impairment in cognitive behavior but an increase in expression of proinflammatory AGEs and their receptor, accompanied by changes in gut microbiota composition (Li et al., 2020). Specifically, *Lactobacillus plantarum* decreased the expression of hippocampal TLR4 (Mohammed et al., 2020).

**Figure 3:**
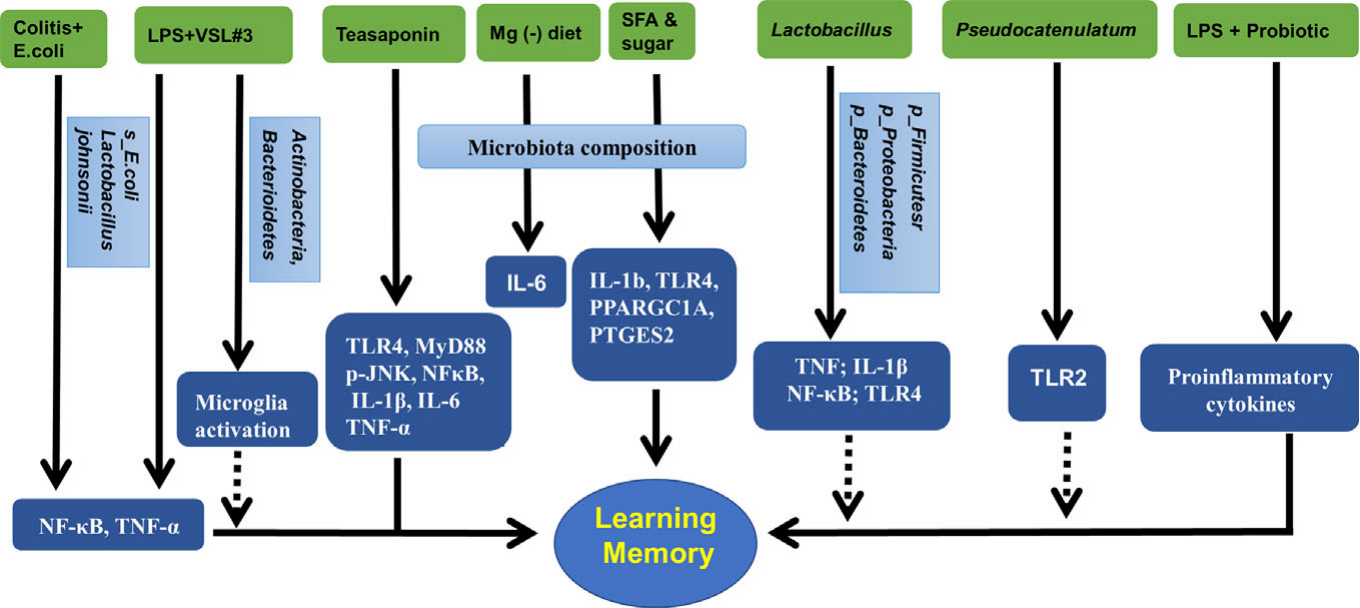
Gut microbiota, hippocampal inflammatory targets, and memory. Mg (-), magnesium deficient diet; SFA, saturated fatty acid; VSL3#, a probiotic mixture comprising 8 Gram-positive bacterial strains; LPS, lipopolysaccharide.

Taken together, the alterations in the gut microbiota may change the inflammatory status in the hippocampus and hippocampus-dependent behaviors, which could be improved by probiotics, microbiota transplantation, or diet management. These results are summarized in **Figure 3** and **Table 4**.

**Table 4:** Gut microbiota and hippocampal inflammatory target.

Treatment	Gut microbiota	Hippocampal inflammatory target	Behavior	References
*Pseudocatenulatum*		TLR2		(Agusti et al., 2018)
magnesium deficient diet	microbiota composition	IL-6		(Winther et al., 2015)
saturated fatty acid and sugar	microbiota PC2 and PC3	IL-1b, TLR4, PPARGC1A, PTGES2	place memory	(Beilharz et al., 2016)
teasaponin	gut microbiota	TLR4, MyD88, p-JNK, NFκB, IL-1β, IL-6, TNF-α	recognition memory	(Wang et al., 2017)
VSL#3	microbiota composition (increase in *Actinobacteria* and *Bacteroidetes*)	CD68, CD11b, microglia activation		(Distrutti et al., 2014)
LPS and FMT	*Firmicutes phylum, Proteobacteria phylum*		spatial memory	(Li et al., 2018)
LPS and probiotic formulation	*Lactobacillus helveticus R0052*, *Bifidobacterium longum R0175*)	proinflammatory cytokines	memory	(Mohammadi et al., 2019)
LPS		NF-κB, microRNA		(Zhao and Lukiw, 2018)
Colitis, *E.coli* and *Lactobacillus johnsonii*	*Enterobacteriaceae, Lactobacillus johnsonii*	NF-κB, TNF-α, LPS	memory behavior	(Jang et al., 2018)
*Lactobacillus brevis OW38*	*Firmicutes* or *Proteobacteria* to *Bacteroidetes* ratio	TNF, IL-1β, NF-κB; LPS		(Jeong et al., 2016)
FMT	microbiota composition	pro-inflammatory AGEs and their receptor	cognitive behavior	(Li et al., 2020)
*Lactobacillus Plantarum*		TLR4		(Mohammed et al., 2020)

PC, microbiota principal component; FMT, fecal microbiota transplantation; LPS, lipopolysaccharide; VSL#3, a probiotic mixture comprising 8 Gram-positive bacterial strains.

## Gut Microbiota and Hippocampal Alzheimer’s Disease Pathologies

Human microbiota may strongly influence the pathology of AD, the deposition of Aβ, and formation of neurofibrillary tangles in the hippocampus (Kohler et al., 2016). The effects of aging and the risk of neurodegenerative diseases can be reduced by probiotics, or by combining probiotics and prebiotics known as synbiotics, which can significantly modify the composition of the gut microbiome (Lye et al., 2018). Long-term (6 months) antibiotic treatment of 2-week-old AD mice induced shifts in gut microbial composition and diversity, a decrease in Aβ plaque deposition, but an increase in soluble Aβ in the brain of AD mice, suggesting that gut microbiota diversity could regulate host innate immunity mechanisms that are related to Aβ amyloidosis (Minter et al., 2016). Moreover, early postnatal (days 14–21) antibiotic treatment resulted in long-term alterations in gut microbial genera (predominantly *Lachnospiraceae* and *S24-7*) and reduced brain Aβ deposition in aged AD mice, accompanied by reduced plaque-localized microglia and astrocytes (Minter et al., 2017). A recent study showed that when 3xTg-AD mice in the early stage of AD were treated with the SLAB51 probiotic formulation, the gut microbiota and their metabolites changed significantly, and the impaired neuronal proteolytic pathways (the ubiquitin proteasome system and autophagy) were partially recovered. Cognitive function improved and the accumulation of Aβ aggregates was reduced (Bonfili et al., 2017).

In APP/PS1 mice, quercetin treatment increased gut microbial diversity and relative abundance of *Glutamicibacter*, *Facklamia*, and *Aerocorrus*; it also improved learning and memory in the Morris water maze test. Hippocampal BDNF levels were increased but Aβ plaques and p-Tau decreased; further analysis revealed that hippocampal p-Tau at ser396 was negatively correlated with *Aerococcus*, but p-Tau at ser404 was negatively correlated with *Facklamia* (Lv et al., 2018). Curcumin has also been shown to improve spatial learning and memory abilities and reduce Aβ plaque in the hippocampus of APP/PS1 mice. These changes may be related to the altered abundance of *Bacteroidaceae*, *Prevotellaceae*, *Lactobacillaceae*, and *Rikenellaceae* at the family level, and *Prevotella*, *Bacteroides*, and *Parabacteroides* at the genus level (Sun et al., 2020). Additionally, as mentioned above, the administration of TTK extract and Tibetan fermented milk also improved memory loss and reduced the deposition of hippocampal Aβ that involved changes in gut *Clostridiales*, *Erysipelotrichales*, *Desulfovibrionales*, *Lactobacillales*, *Bacteroides*, *Faecalibacterium* spp. Mucispirillum, and *Ruminiclostridium* (Kim et al., 2020; Liu et al., 2020). Additionally, mice treated with a ketogenic diet for 16 weeks showed significantly increased abundance of putatively beneficial gut microbiota (*Akkermansia muciniphila* and *Lactobacillus*), and reduced putatively proinflammatory taxa (*Desulfovibrio* and *Turicibacter*). These changes facilitated the clearance of Aβ, and reduced the risk of AD (Ma et al., 2018). Moreover, oral administration of grape seed polyphenol extract (GSPE) resulted in an increase in two phenolic acids, 3-hydroxybenzoic acid and 3-(3-hydroxyphenyl) propionic acid in rats. This treatment also interfered with the assembly of Aβ peptides into senile plaques, suggesting an important contribution of the intestinal microbiota to the protective activities of GSPE in AD (Wang et al., 2015). In a population-based cross-sectional cohort study, a very intriguing discovery was that the Mediterranean diet, which contains an unusually large quantity of *Lactobacilli*, seemed very effective in preventing AD (Jin et al., 2018). Furthermore, it has been reported that in APP/PS1mice, prebiotic fructooligosaccharide (FOS) treatment altered microbial composition, ameliorated cognitive deficits and AD pathological changes, and upregulated the expression levels of hippocampal synaptic proteins (Sun et al., 2019). Similar results were also detected in other species. When AD rats were treated with FOS from *Morinda officinalis*, the learning and memory abilities were significantly ameliorated, accompanied with maintenance of the diversity and stability of the gut microbial community (Chen et al., 2017). Interestingly, a recent study revealed that gut microbiota diversity and composition might also mediate the effects of chronic noise exposure on cognitive impairment and hippocampal Aβ deposition, and microbiota transplantation demonstrated that the host impairment of epithelial integrity and AD-like changes were driven by the noise exposure-altered microbiota (Cui et al., 2018).

**Figure 4:**
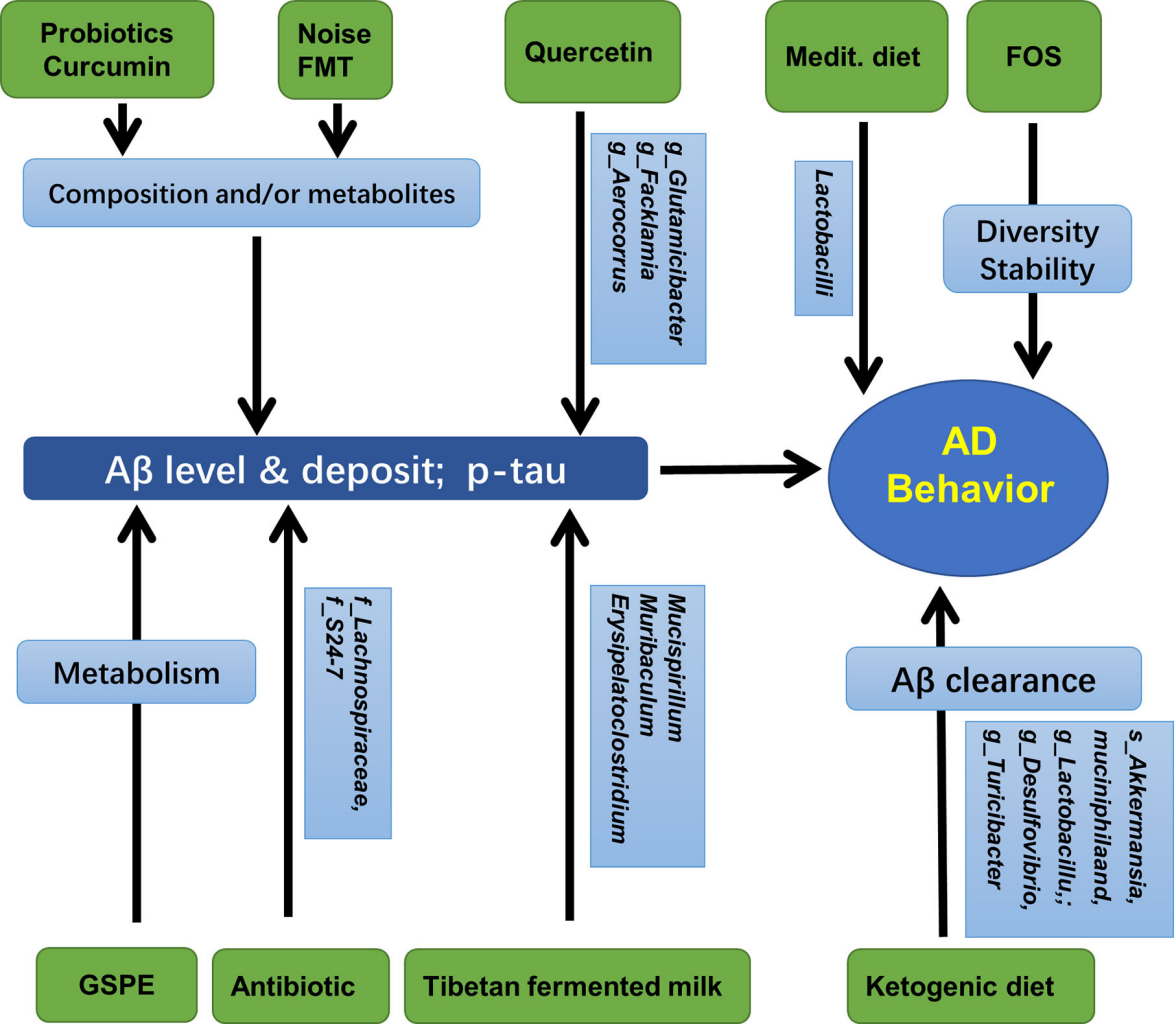
Gut microbiota, hippocampal AD pathology, and AD behaviors. Medit, Mediterranean diet; GSPE, Grape seed polyphenol extract; FOS, fructooligosaccharides; FMT, fecal microbiota transplantation.

Taken together, as reviewed by Sun et al. (Sun et al., 2020), the composition and diversity of gut microbiota may be regulated in many ways, such as antibiotics, probiotics, diet, plant extracts, and microbiota transplantation. These treatments were also shown to be deeply involved in AD pathology, especially the formation and deposition of Aβ, and behaviors. These results are summarized in **Figure 4** and **Table 5**.

**Table 5:** Gut microbiota affects AD pathology and behaviors.

Treatment	Gut microbiota	Hippocampal target	Pathology/Behavior	References
antibiotic	composition and diversity	microglia, Aβ		(Minter et al., 2016)
antibiotic	*Lachnospiraceae* and *S24-7*	microglia, astrocyte, Aβ		(Minter et al., 2017)
SLAB51 probiotic formulation	Composition and metabolites	Aβ deposit, ubiquitin proteasome system and autophagy	cognition (open field, novel object recognition, passive avoidance, elevated plus maze)	(Bonfili et al., 2017)
quercetin	*Glutamicibacter*, *Facklamia Aerocorrus*	BDNF, Aβ deposit, p-tau	learning and memory	(Lv et al., 2018)
curcumin	*Bacteroidaceae*, *Prevotellaceae*, *Lactobacillaceae*, *Rikenellaceae*	Aβ deposit	spatial learning and memory	(Sun et al., 2020)
TTK extract and Tibetan fermented milk	*Clostridiales*, *Erysipelotrichales*, *Desulfovibrionales*, *Lactobacilales, Bacteroides*, *Faecalibacterium* spp. Mucispirillum, *Ruminiclostridium*	Aβ deposit	spatial learning and memory	(Kim et al., 2020) (Liu et al., 2020) (Kim et al., 2020; Liu et al., 2020)
ketogenic diet	*Akkermansia* muciniphila, *Lactobacillus*; *Desulfovibrio*, *Turicibacter*	Aβ clearance		(Ma et al., 2018)
Mediterranean diet	*Lactobacilli*		AD -preventing	(Jin et al., 2018)
grape seed polyphenol extract	microbiota metabolism	Aβ deposit		(Wang et al., 2015)
noise/microbiota transplantation	composition and diversity	Aβ deposit	learning and memory	(Cui et al., 2018)
FOS	microbial composition	AD pathology, synaptic plasticity	cognition (open field, Morris water maze, object recognition)	(Sun et al., 2019)
FOS	diversity and stability		learning and memory	(Chen et al., 2017)

SLAB51, a formulation made of nine live bacterial strains [Streptococcus thermophilus, bifidobacteria (B. longum, B. breve, B. infantis), lactobacilli (L. acidophilus, L plantarum, L. paracasei, L. delbrueckii subsp. bulgaricus, L. brevis)]; TTK, Tetragonia tetragonioides Kuntze; FOS, prebiotic fructooligosaccharides.

## Clinical Applications of Probiotics and Antibiotic on Brain Cognitive Function

Limited clinical trials have addressed the effects of probiotics on brain function, including memory, depression, and stress. Steenbergen et al. reported that multispecies probiotic intervention could reduce negative thoughts associated with a sad mood in healthy volunteers (Steenbergen et al., 2015). Later, probiotic administration was shown to alter brain activities related to emotional memory, decision-making tasks, anxiety, negative affect, and worry, which were also accompanied by subtle shifts in the gut microbiome profile (Bagga et al., 2018; Tran et al., 2019). *Bifidobacterium longum 1714™* also modulated the resting neural activity in several brain regions including the hippocampus, fusiform, and temporal cortex, which correlated with enhanced vitality and reduced mental fatigue in healthy volunteers during social stress (Wang et al., 2019). Inoue et al. reported that probiotic *bifidobacteria* supplementation showed stronger effects on the improvement of mental condition compared to moderate resistance training (Inoue et al., 2018).

Probiotics have also been shown to be effective in patients with cognitive disorders. In patients with mild cognitive impairment, treatment with *Lactobacillus plantarum C29*-fermented soybean supplement (DW2009) resulted in significant improvement in cognitive function (Hwang et al., 2019). For major depressive patients, probiotics alone or in combination with antidepressants are effective and well tolerated (Miyaoka et al., 2018; Chahwan et al., 2019). Similarly, probiotic *Lactobacillus plantarum 299v* decreased kynurenine concentration and improved cognitive functions in patients with major depression (Rudzki et al., 2019). The probiotic *Lactobacillus plantarum P8* gender-dependently alleviated stress and enhanced memory and cognition, such as social emotional cognition, and verbal learning and memory (Lew et al., 2019).

In peripheral disorders, probiotics and antibiotics may affect brain function through regulation of microbiota. Probiotic *Bifidobacterium Longum NCC3001* administration has also been shown to reduce depression and alter brain activity in patients with irritable bowel syndrome (Pinto-Sanchez et al., 2017), improve impulsivity and decision-making in patients with fibromyalgia (Roman et al., 2018), and neurocognitive functions in human immunodeficiency virus transfected patients (Ceccarelli et al., 2017). Rifaximin is a gut-specific antibiotic. Several clinical trials demonstrated that in patients with minimal hepatic encephalopathy (MHE), rifaximin induced a significant improvement in cognition, including working memory that involved *Enterobacteriaceae*, *Porphyromonadaceae*, and *Bacteroidaceae*, endotoxemia, and several serum fatty acids. This treatment also decreased *Veillonellaceae* and increased *Eubacteriaceae*, inducing a shift from pathogenic to beneficial metabolite linkages (Bajaj et al., 2013; Ahluwalia et al., 2014). Additionally, in patients with MHE, oral capsular FMT (enriched in *Lachnospiraceae* and *Ruminococcaceae*) improved cognition. Inflammation was positively correlated with greater complexity of beneficial taxa, such as *Ruminococcaceae*, *Verrucomicrobiaceae*, and *Lachnospiraceae*; increased duodenal mucosal diversity with higher *Ruminococcaceae* and *Bifidobacteriaceae;* and lower *Streptococcaceae* and *Veillonellaceae*, indicating the beneficial effects of capsular FMT on inflammation and cognition in patients with cirrhosis (Bajaj et al., 2019a; Bajaj et al., 2019). The above results were summarized in **Table 6**.

**Table 6:** Clinical trials on gut microbiota and hippocampus-dependent behaviors.

Treatment	Gut microbiota	Pathology/Behavior	References
multispecies probiotics^1^		sad mood-related negative thoughts	(Steenbergen et al., 2015)
probiotic administration (Ecologic^®^825, etc.)	diversity and composition (*Bacteroides etc*.)	emotional memory, decision-making tasks, anxiety, negative affect and worry	(Bagga et al., 2018; Tran et al., 2019)
*Bifidobacterium longum* 1714™		social stress	(Wang et al., 2019)
*bifidobacteria* supplementation^2^		mental condition	(Inoue et al., 2018)
DW2009	*lactobacilli* population	cognitive functions	(Hwang et al., 2019)
Probiotics^3^		major depressive disorder	(Miyaoka et al., 2018; Chahwan et al., 2019)
*Lactobacillus Plantarum* 299v		major depression-related cognitive functions	(Rudzki et al., 2019)
*Lactobacillus plantarum* P8		stress, memory, and cognition (social emotional cognition and verbal learning and memory)	(Lew et al., 2019)
*Bifidobacterium Longum* NCC3001		depression and brain activity, impulsivity, and decision-making	(Ceccarelli et al., 2017; Pinto-Sanchez et al., 2017; Roman et al., 2018)
Rifaximin	*Enterobacteriaceae*, *Porphyromonadaceae*, *Bacteroidaceae*, *Veillonellaceae*, *Eubacteriaceae*	working memory	(Bajaj et al., 2013; Ahluwalia et al., 2014)
FMT (enrich in Lachnospiraceae and Ruminococcaceae)	*Ruminococcaceae*, *Verrucomicrobiaceae*, *Lachnospiraceae*, *Ruminococcaceae*, *Bifidobacteriaceae*, *Streptococcaceae*, *Veillonellaceae*	cognition and inflammation	(Bajaj et al., 2019a; Bajaj et al., 2019b).

1: Bifidobacterium bifidum W23, Bifidobacterium lactis W52, Lactobacillus acidophilus W37, Lactobacillus brevis W63, L. casei W56, Lactobacillus salivarius W24, Lactococcus lactis (W19 and W58)

2: B. longum BB536, B. infantis M-63, B. breve M-16V and B.breve B-3;

3: Clostridium butyricum MIYAIRI 588; Bifidobacterium bifidum W23, Bifidobacterium lactis W51, Bifidobacterium lactis W52, L. acidophilus W37, Lactobacillus brevis W63, Lactobacillus casei W56, Lactobacillus salivarius W24, Lactococcus lactis W19 and Lactococcus lactis W58;

Ecologic^®^825 contains nine bacterial strains: Lactobacillus casei W56, Lactobacillus acidophilus W22, Lactobacillus paracasei W20, Bifidobacterium lactis W51, Lactobacillus salivarius W24, Lactococcus lactis W19, Bifidobacterium lactis W52, Lactobacillus plantarum W62, and Bifidobacterium bifidum W23.

DW2009, Lactobacillus plantarum C29-fermented soybean supplement; FMT, fecal microbiota transplantation.

## Conclusions

**Figure 5:**
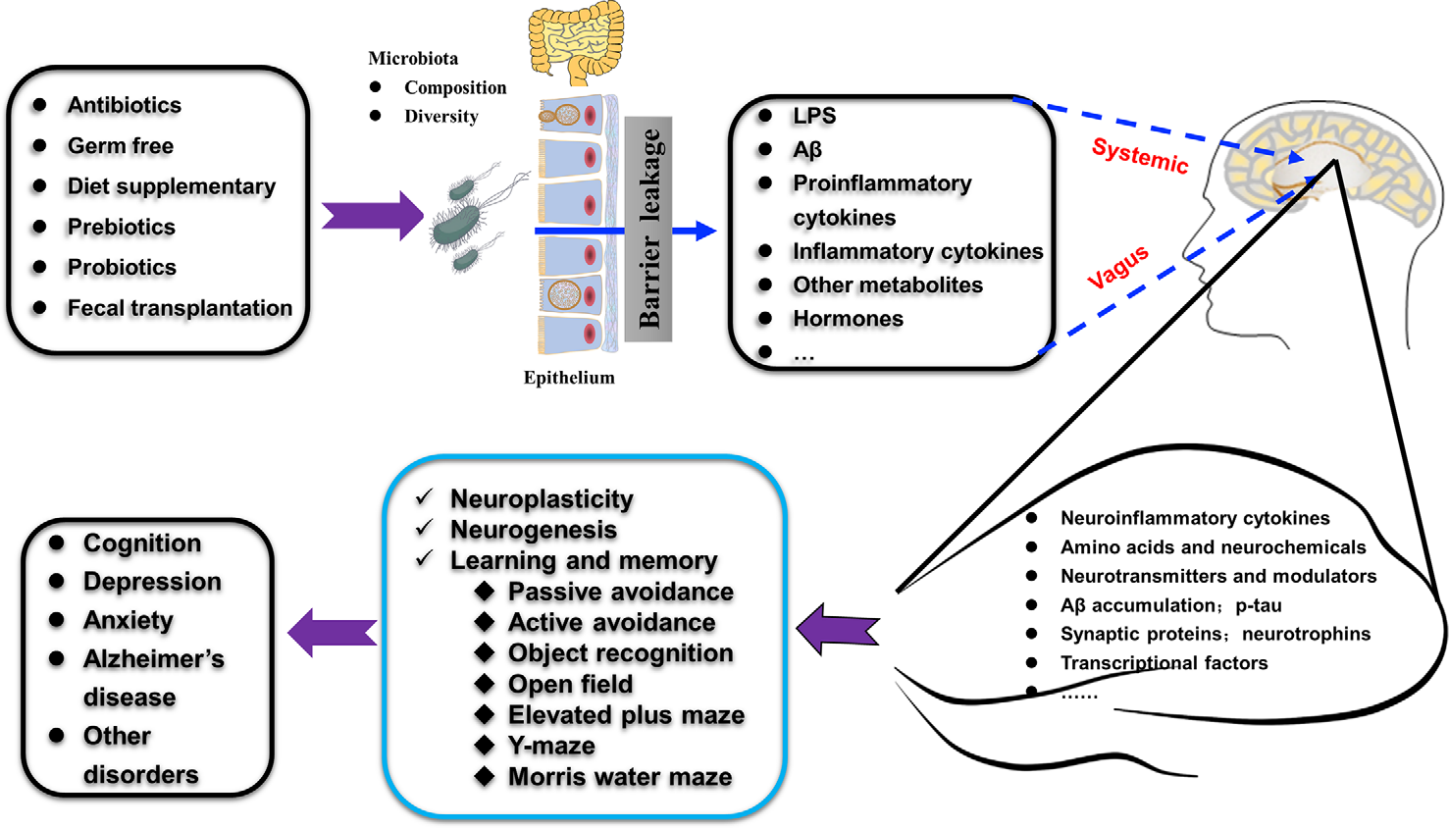
Schematic illustration shows how gut microbiota affect hippocampal plasticity and behaviors through gut-hippocampus-axis. The composition and diversity, the levels of LPS and Aβ, proinflammatory and inflammatory factors, and other metabolites (such as hormones) of gut microbiota could be affected by many treatments (antibiotics, germ free administration, diet, pre- and pro-biotics, and fecal transplantation). The alterations occurred in the gut microbiota affect many aspects of the hippocampus (such as amino acids, expression of specific proteins, AD-related pathologies) through the vagus nerve pathway, the systemic pathway (with the release of hormones, metabolites, cytokines, and neurotransmitters) to increase the permeability of mucosa-intestinal barrier and blood-brain barrier and finally regulate hippocampus-dependent cognition and behaviors.

The gut microbiota is regarded as the second genome of the human body. Its composition and diversity changes frequently under different conditions. The hippocampus is the center for learning and memory, which is closely related to dementia and many other mental disorders. In this manuscript, we reviewed recent findings on the relationship between intestinal microbes and the plasticity, neurochemicals, and function of the hippocampus. We highlighted the advances in modulating hippocampal structure and behavior using probiotics, prebiotics, and diet through the gut microbiota-hippocampus axis, as summarized in **Figure 5**.

Evidence indicates that the gut microbiota is altered in AD. Therefore, modifying the gut microbiota may affect this disease (Agahi et al., 2018). An abundance of “good bacteria” such as *Bifidobacterium* or their products have generally been believed to be beneficial, while “bad bacteria” such as *Clostridium* are assumed to be detrimental (Park et al., 2017). *Escherichia coli* and *Salmonella enterica* are among the many bacterial strains that express and secrete Aβ and contribute to AD pathogenesis (Tse, 2017). Clinical studies have shown that, in cognitively impaired elderly patients with brain amyloidosis, the anti-inflammatory species *Eubacterium rectale* and *Bacteroides fragilis* were more abundant, while proinflammatory genera such as *Escherichia/Shigella* were higher. Supplementation with *Lactobacilli*- and *Bifidobacteria*-based probiotics was neuroprotective in AD subjects (Mancuso and Santangelo, 2018). However, the results of current studies are controversial. For example, Vogt et al. reported an increase in the abundance of *Bacteroidaceae*, *Rikenellaceae*, and *Gemellaceae*, but a decrease in that of *Ruminococcaceae*, *Bifidobacteriaceae*, *Clostridiaceae*, *Mogibacteriaceae*, *Turicibacteraceae*, and *Peptostreptococcaceae* in AD patients when compared with the controls (Vogt et al., 2017); Zhuang et al. reported an increase in the abundance of *Ruminococcaceae*, *Enterococcaceae*, and *Lactobacillaceae*, but a decrease in that of *Lanchnospiraceae*, *Bacteroidaceae*, and *Veillonellaceae* compared with the control group (Zhuang et al., 2018).

The exact trigger of AD remains unknown. Current treatments for AD are limited, and great efforts have been made to target Aβ plaques, but these attempts have often ended in failure (Reiss et al., 2018; Salminen et al., 2018). Recent progress in the effects of gut microbiota on hippocampus-dependent learning and memory have opened a new window for understanding the onset and progression of AD. Thus, modulation of the gut microbiota has been regarded as a preventive and therapeutic target against this worldwide challenge. However, how the gut microbiota affects the structure and function of the hippocampus is far from clear. It has been shown that bacterial metabolites, such as LPS and Aβ, may act through the vagus nerve pathway, the systemic pathway (with the release of hormones, metabolites, and neurotransmitters), and the immune pathway (by the action of cytokines) to increase the permeability of the mucosa-intestinal barrier and blood-brain barrier, induce hippocampal inflammation, and ultimately affect hippocampus-dependent functions (Bostanciklioglu, 2019; Garcez et al., 2019; Gomez-Eguilaz et al., 2019). All these still require further experimental evidence, and we also lack human observational or interventional data to propose any clinical recommendations.

## Author Contributions

YY and DC conceived this article. WT and ZM performed the literature search, data analysis, and draft preparation. YY and DC critically revised the manuscript. NL, YL, and LL helped in the data analysis and draft preparation. All authors contributed to the article and approved the submitted version.

## Funding

This study was supported in part by the National Natural Science Foundation of China (NSFC 81500408 to YY and NSFC 81972305 to DC), Foundation and Frontier Projects of Chongqing Science and Technology Commission (Grant No. cstc2016jcyjA0079 to YY), and China Postdoctoral Science Foundation Grant (2019M653976).

## Conflict of Interest

The authors declare that the research was conducted in the absence of any commercial or financial relationships that could be construed as a potential conflict of interest.

## Abbreviations

AD, Alzheimer’s disease; AGEs, advanced glycation end products; Aβ, β-amyloid; *B. breve* A1, *Bifidobacterium breve strain* A1; BDNF, brain-derived neurotrophic factor; *C. butyricum*, *Clostridium butyricum*; CA, cornu ammonis (hippocampus); Caf, cafeteria diet; CREB, cAMP-response element-binding protein; DG, dentate gyrus; DW2009, *Lactobacillus plantarum C29*-fermented soybean supplement; FMT, fecal microbiota transplantation; FOS, fructooligosaccharides; GF, germ free; GSPE, grape seed polyphenol extract; HC, healthy control; IL-6, interleukin-6; LJ, *Lactobacillus johnsonii* CJLJ103; lncRNAs, long non-coding RNAs; LPS, lipopolysaccharide; LW-AFC, an herbal medicine prepared from traditional Chinese medicine LiuweiDihuang decoction; MHE, minimal hepatic encephalopathy; OTUs, operational taxonomic units; PC, microbiota principal component; pCREB, phosphorylation CREB; p-Tau, phospho-tau protein; SPF, specific pathogen free; TLR, Toll-like receptor; TTK, Tetragonia tetragonioides Kuntze; VSL#3, a probiotic mixture comprising 8 Gram-positive bacterial strains; WT, wild type.
